# A Qualitative Market Analysis Applied to Mini-FLOTAC and Fill-FLOTAC for Diagnosis of Helminth Infections in Ruminants

**DOI:** 10.3389/fvets.2020.580649

**Published:** 2020-10-22

**Authors:** Maria Paola Maurelli, Oliva Maria Dourado Martins, Eric R. Morgan, Johannes Charlier, Giuseppe Cringoli, Teresa Letra Mateus, Bogdan Bacescu, Christophe Chartier, Edwin Claerebout, Theo de Waal, Christina Helm, Hubertus Hertzberg, Barbara Hinney, Johan Höglund, Iveta Angela Kyriánová, Marcin Mickiewicz, Saulius Petkevičius, Stanislav Simin, Smaragda Sotiraki, Marina Tosheska, Mariann Toth, María Martínez-Valladares, Marian Varady, Blagica Sekovska, Georg von Samson-Himmelstjerna, Laura Rinaldi

**Affiliations:** ^1^Department of Veterinary Medicine and Animal Production, University of Naples Federico II, Naples, Italy; ^2^Ci2, Polytechnic Institute of Tomar, Tomar, Portugal; ^3^Institute of Global Food Security, Queen's University Belfast, Belfast, United Kingdom; ^4^Kreavet, Kruibeke, Belgium; ^5^CISAS—Centre for Research and Development in Agrifood Systems and Sustainability, Escola Superior Agrária, Instituto Politécnico de Viana do Castelo, Rua Escola Industrial e Comercial de Nun'Àlvares, Viana do Castelo, Portugal; ^6^EpiUnit—Instituto de Saúde Pública da Universidade do Porto, Porto, Portugal; ^7^Faculty of Veterinary Medicine, Spiru Haret University, Bucharest, Romania; ^8^INRA, Oniris, BIOEPAR, Nantes, France; ^9^Laboratory for Parasitology, Faculty of Veterinary Medicine, Ghent University, Merelbeke, Belgium; ^10^School of Veterinary Medicine, University College Dublin, Dublin, Ireland; ^11^Institute for Parasitology and Tropical Veterinary Medicine, Freie Universitaet Berlin, Berlin, Germany; ^12^Institute of Parasitology, University of Zurich, Zurich, Switzerland; ^13^Institute of Parasitology, Vetmeduni Vienna, Vienna, Austria; ^14^Department of Biomedical Sciences and Veterinary Public Health, Section for Parasitology Swedish University of Agricultural Sciences, Uppsala, Sweden; ^15^Faculty of Agrobiology, Food and Natural Resources, Czech University of Life Sciences Prague, Prague, Czechia; ^16^Division of Veterinary Epidemiology and Economics, Institute of Veterinary Medicine, Warsaw University of Life Sciences, Warsaw, Poland; ^17^Lithuanian University of Health Sciences, Kaunas, Lithuania; ^18^Department of Veterinary Medicine, Faculty of Agriculture, University of Novi Sad, Novi Sad, Serbia; ^19^Veterinary Research Institute, HAO-DEMETER, Thessaloniki, Greece; ^20^Lag Agro Lider, Prilep, North Macedonia; ^21^Institutes of Agricultural Research and Educational Farm, Research Institute of Karcag, University of Debrecen, Debrecen, Hungary; ^22^Instituto de Ganadería de Montaña, CSIC-Universidad de León, León, Spain; ^23^Institute of Parasitology of the Slovak Academy of Sciences, Košice, Slovakia; ^24^Faculty of Veterinary Medicine, St. Cyril and Methodius University, Skopje, North Macedonia

**Keywords:** Mini-FLOTAC, Fill-FLOTAC, anthelmintic resistance (AR), SWOT analysis, PESTEL analysis

## Abstract

Helminth infections, mainly by gastrointestinal nematodes (GIN), are one of the main concerns for animal health, welfare and productivity in grazing ruminant livestock worldwide. The use of a sensitive, precise, accurate, low-cost, and easy-to-perform copromicroscopic technique is of pivotal importance to perform reliable fecal egg count (FEC) and fecal egg count reduction test (FECRT), in order to determine the need of anthelmintic treatment, but also anthelmintic efficacy or resistance. This approach is fundamental to a correct and efficient control of GIN. Unfortunately, in worldwide ruminant farm practice, repeated anthelmintic treatments are carried out, without prior diagnosis of infection, contributing to the spread of Anthelmintic Resistance (AR). Tackling this phenomenon, improving mainly the GIN diagnosis and AR status in farm animals, is a priority of the European COST Action “COMBAR—COMBatting Anthelmintic Resistance in Ruminants” and of the STAR-IDAZ International Research Consortium on Animal Health. One of the specific objectives of the COMBAR Working Group 1 (WG1) is to conduct an European market analysis of new diagnostics and develop a business plan for commercial test introduction, leveraging technical know-how of participants. Since the Mini-FLOTAC in combination with the Fill-FLOTAC may be considered a good candidate for a standardized FEC and FECRT in the laboratory, as well as directly in the field, the aim of this study was to conduct SWOT (Strength—Weaknesses—Opportunities—Threats) and PESTEL (Political, Economic, Social, Technological, Environmental, and Legal) analyses of these tools in 20 European countries involved in the COMBAR WG1, in order to identify the opportunities, barriers, and challenges that might affect the Mini-FLOTAC and Fill-FLOTAC commercialization in Europe.

## Introduction

Helminth infections, mainly by gastrointestinal nematodes (GIN), are one of the main concerns for animal health, welfare and productivity in grazing ruminant livestock worldwide (1, 2). In ruminant farm practice, the control of GIN is usually carried out using repeated and sometimes blind anthelmintic treatments, without prior diagnosis of infection. This approach has contributed to the spread of anthelmintic resistance (AR) which is reported worldwide in multiple GIN species, especially in sheep, against all commercially available anthelmintic classes (3–7). Tackling this phenomenon is a priority of the European COST Action “COMBAR—COMBatting Anthelmintic Resistance in Ruminants” (https://www.combar-ca.eu/) and of the STAR-IDAZ International Research Consortium on Animal Health (https://www.star-idaz.net/). One of the main goals of these networks is to improve diagnosis of GIN and AR status in farm animals. Therefore, the use of reliable, low-cost, easy-to-perform fecal egg count (FEC) methods is of pivotal importance to quantify GIN eggs in fecal samples, in order to determine the need of anthelmintic treatment, anthelmintic efficacy or resistance, through the widely used fecal egg count reduction test (FECRT) (7).

**Figure 1:**
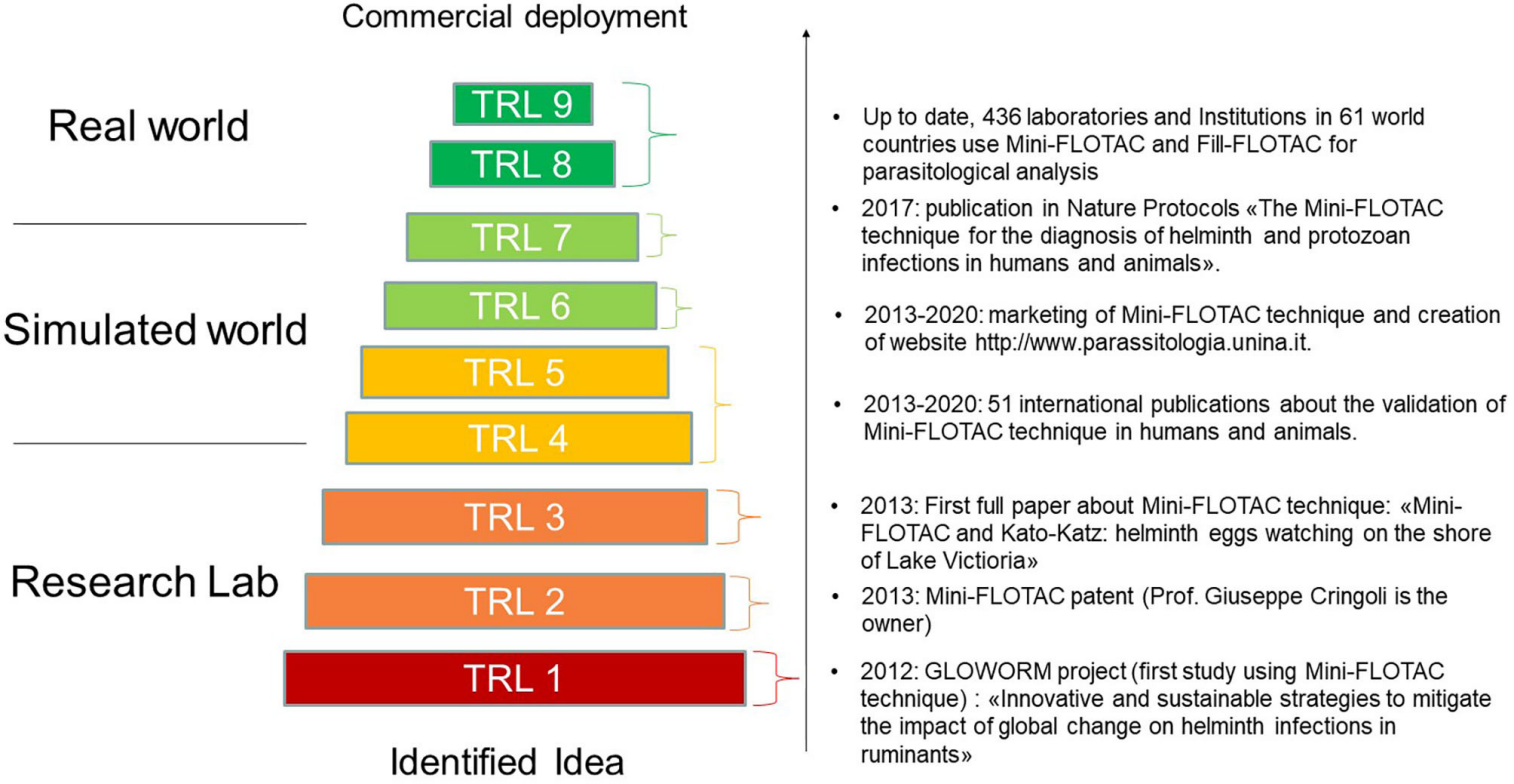
TRL of the Mini-FLOTAC technique.

Mini-FLOTAC and Fill-FLOTAC, developed by the Unit of Parasitology and Parasitic Diseases of the Department of Veterinary Medicine and Animal Production (University of Naples Federico II), are easy-to-use devices, used in combination to perform the Mini-FLOTAC technique, a multivalent, sensitive, accurate, precise, and reproducible copromicroscopic method (8). Because of these characteristics, these tools have increasingly been employed in FEC and FECRT surveys (9–18). The Mini-FLOTAC is a self-contained device for viewing and counting helminth eggs per gram of feces, under the microscope, after preparation from fecal samples, using the Fill-FLOTAC device (8). Over 50 scientific publications on the validation of the Mini-FLOTAC and Fill-FLOTAC have been published in international journals having a high citation index, whilst the Standard Operating Procedures of the technique have been published in Nature Protocols (8). Thus, the Mini-FLOTAC technique can be situated at level 9 on the Technology Readiness Level scale (Figure 1). These devices are today distributed free of charge, without profit by the University of Naples (www.parassitologia.unina.it), although an economic contribution is required to cover costs of production and packaging. To date this method has been adopted by over 400 laboratories worldwide for research purposes, as well as for routine diagnosis of helminth infections and other parasites.

The general objective of the COMBAR Working Group 1 (WG1) is to prioritize, evaluate and implement cost-effective methods for the diagnosis of helminth infections and AR. Furthermore, one of the specific objectives is to conduct an European market analysis of new diagnostics and develop a business plan for commercial test introduction, leveraging technical know-how of participants. Since the Mini-FLOTAC technique may be considered a good candidate for a standardized FEC and FECRT, the aim of this study was to conduct SWOT (Strength—Weaknesses—Opportunities—Threats) and PESTEL (Political, Economic, Social, Technological, Environmental, and Legal) analyses of the Mini-FLOTAC and Fill-FLOTAC in 20 European countries involved in the COMBAR WG1, in order to identify the opportunities, barriers and challenges that might affect their commercialization in Europe.

## Materials and Methods

### SWOT Analysis

The research was developed in three phases. In the first phase, a SWOT analysis was performed, based on the scientific literature on Mini-FLOTAC and Fill-FLOTAC, considering in detail the positive and negative, internal (strength and weakness) and external (opportunities and threats) factors that could affect their commercialization in Europe.

### PESTEL Analysis in Italy and Other European Countries

In the second and third phases a qualitative macro analysis of the negative and positive aspects of the PESTEL factors (Table 1) that could impact on the commercialization of the Mini-FLOTAC and Fill-FLOTAC was carried out in 20 European countries: Austria, Belgium, Czechia, France, Germany, Greece, Hungary, Ireland, Italy, Lithuania, North Macedonia, Poland, Portugal, Romania, Republic of Serbia, Spain, Slovakia, Sweden, Switzerland, and the United Kingdom (UK). We decided to conduct a qualitative analysis, because it is very useful to explore new factors, as in this study, involving an iterative process, with the aim to better understand how or why a phenomenon occurs (19). Another added value of this approach is the ability of a researcher to explore different details, without having to confirm or deny hypotheses (20).

**Table 1:** PESTEL factors analyzed for the Mini-FLOTAC and Fill-FLOTAC and example sources.

**PESTEL factors**	**Sources**
Political	e.g., Government statement
Economic	e.g., Statistic report
Social	e.g., Government report
Technological	e.g., Organization report
Environmental	e.g., Non-profit report
Legal	e.g., Law number

In detail, during the second phase, the researchers at the Unit of Parasitology and Parasitic Diseases of the Department of Veterinary Medicine and Animal Production, University of Naples Federico II (PAR-UNINA), who best know this device, were interviewed on the PESTEL factors that could affect the Mini-FLOTAC and Fill-FLOTAC commercialization in Italy (Table 1). The PAR-UNINA researchers were chosen for their three complementary dimensions of competences: (i) theoretical, because they are the inventors and developers of the Mini-FLOTAC technique; (ii) empirical and (iii) practical, according to their pluriannual experience on the use of the devices for FEC and FECRT of GIN in livestock.

Finally, in the third phase, the same PESTEL analysis was conducted in all the 20 countries involved in the COMBAR WG1, to identify the barriers and challenges for the commercialization of the Mini-FLOTAC and Fill-FLOTAC in Europe. The questionnaire was available online, from September 26, 2019 to April 10, 2020 at the link https://survey.zohopublic.eu/zs/8FB8NY. The participants were parasitologists with a long-term experience on the use of traditional copromicroscopic techniques, as well as those that have been using the Mini-FLOTAC technique for at least 3 years.

The qualitative analysis, in this phase, aimed to obtain, in a neutral way, the opinions and experiences of the respondents (21).

Moreover, a pragmatic research approach that considered the differences of each country was developed (22).

To reach this purpose, a content analysis of data of the European PESTEL allowed us to identify and organize variables and keywords to evaluate similarities and differences among the countries involved (23, 24). In qualitative research, a high level of transparency is crucial to better understand the relevance of these data among countries (25). In our study, this principle was assured, because all the researchers involved knew the aim of the study, being the European market analysis of new diagnostics, one of the deliverables of the COMBAR WG1.

## Results and Discussion

The results are presented according to the phases of the research: (i) the SWOT analysis; (ii) the PESTEL analysis in Italy; (iii) the PESTEL analysis in Europe.

### SWOT Analysis

The main results of the analysis of the key internal and external factors that could influence the distribution and commercialization of the Mini-FLOTAC and Fill-FLOTAC are reported in Table 2.

**Table 2:** SWOT analysis of the Mini-FLOTAC and Fill-FLOTAC, which are used in combination to accurately estimate helminth egg density in fecal samples.

**Internal factors**	**External factors**
**Strengths**	**Opportunities**
Multivalent technique: applicable to multiple parasites in different host species	Increasing demand for standardized diagnostic devices for FEC and FECRT
High specificity, sensitivity, accuracy, precision, reproducibility, and repeatability	Interest from research institutes, universities, diagnostic laboratories, veterinary clinics, veterinarians
No requirements of special equipment or specialized technicians (user-friendly)	Easy distribution with all the information about the product available on the website www.parassitologia.unina.it
Pen-side use (practical for on-farm use)	Advice (before and after the purchase)
Possibility to process pooled fecal samples	Tutorials to perform the Mini-FLOTAC techniques
Possibility to preserve fecal samples	Free residential courses
Closed system: minimizes spillage and protects hygiene and operator health	Support to veterinary associations, farmer associations, and veterinary clinics
Low cost (€15 for Mini-FLOTAC+ €10 for Fill-FLOTAC)	Development of an automated system to reduce human errors and time of analysis
Re-usable devices (Mini-FLOTAC = up to 50 times, Fill-FLOTAC up to 200 times)	Possibility to become local distributor
Eco-friendly	
**Weakness**	**Threats**
Influence of the preservation method of sample	Barriers in adopting new techniques for lab technicians
Influence of the flotation solution	Pressure to keep a low price in the veterinary sector
If you use formalin to fix the fecal samples, special precautions have to be adopted	Customer may be misinformed or influenced by external factors
Operator-dependent technique; technicians able to recognize parasitic elements	Possibility of being copied
Other techniques are to be preferred to detect eggs of trematodes, if their number is very low	
The only distributor in Europe is PAR-UNINA	

The most important aspects of the SWOT analysis are reported below.

#### Strengths

**Figure 2:**
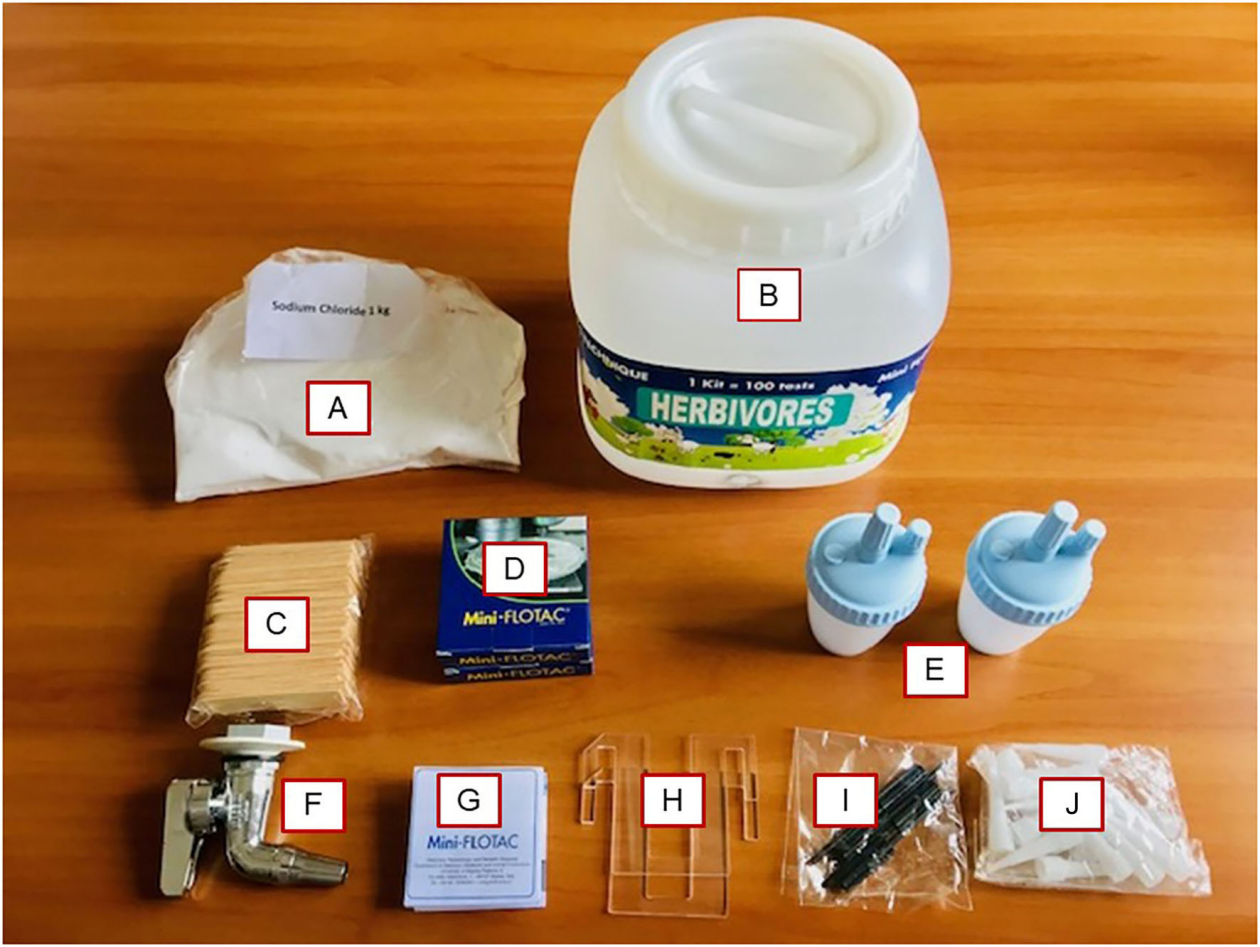
Mini-FLOTAC Kit 100 tests for field use. **(A)** Salt for Flotation Solution; **(B)** Tank; **(C)** Wooden spatula (*n* = 100); **(D)** Mini-FLOTAC (*n* = 2); **(E)** Fill-FLOTAC (*n* = 2); **(F)** Tap; **(G)** Instructions; **(H)** Microscope adaptor (*n* = 2); **(I)** Devices to disassembly Fill-FLOTAC; **(J)** Tips for Fill-FLOTAC.

Mini-FLOTAC is a multivalent technique (i.e., it permits the contemporaneous diagnosis of oocysts and cysts of protozoa, eggs and larvae of nematodes, eggs of trematodes, and cestodes) (8). For this reason, the Mini-FLOTAC and Fill-FLOTAC are able to reduce time and costs of analysis. Moreover, the Mini-FLOTAC technique when compared to other fecal flotation methods showed an overall higher specificity, sensitivity, accuracy, precision, reproducibility, and repeatability for FEC and FECRT of GIN in livestock (9–15, 17). In addition, the Mini-FLOTAC and Fill-FLOTAC are user-friendly devices (i.e., no special equipment such as a centrifuge, or trained technicians are required), so they can be used directly by veterinarians or farmers in the field (pen-side use) (8). Recently, Rinaldi et al. (16) showed that the use on farm of a portable Mini-FLOTAC-kit (composed of 2 Fill-FLOTAC, 2 Mini-FLOTAC, the salt to prepare the flotation solution and all the material necessary to perform the Mini-FLOTAC technique) (Figure 2) and a portable microscope is a swift and cost-effective procedure for FEC of GIN in cattle. To further reduce time and costs, a pooling strategy has been successfully developed and validated for cattle and sheep feces using the Mini-FLOTAC technique (9, 11, 12, 16).

Another important strength of this method is that it can be performed on fresh, but also on preserved fecal samples (e.g., fixed with formalin 5%, for preserving human stool samples). For humans, it is possible to collect the stool sample and add the fixative directly in the Fill-FLOTAC, for processing in subsequent days with an easier planning of work and an efficient quality control in the laboratory (8, 26). However, for livestock feces, it is possible to preserve the samples under vacuum up to 21 days, at +4°C (27) and then analyse them, using the Mini-FLOTAC technique.

Moreover, the combined use of the Fill-FLOTAC and Mini-FLOTAC provides a closed system that minimizes the exposure of the laboratory personnel to potential biohazards (e.g., zoonotic microorganisms in feces or dangerous fixatives) (8).

The total cost of the Mini-FLOTAC + Fill-FLOTAC devices is lower than other competitors (i.e., €15 for the Mini-FLOTAC + €10 for the Fill-FLOTAC vs. €45 for the McMaster, https://vetlabsupplies.co.uk/products/small-equipment/mcmaster-counting-slides/, vs. €600 for the FECPAK, https://www.techion.com/), allowing their wide use in laboratories and on farms.

Finally, the Mini-FLOTAC is re-usable up to 50 times and the Fill-FLOTAC up to 200 times, so they can be considered eco-friendly (8). For the Fill-FLOTAC a biodegradable version is in progress.

#### Weaknesses

The choice of the preservation method of fecal samples (e.g., formalin 5 or 10% or sodium acetate-acetic acid-formalin), the duration of preservation and the choice of the flotation solution can influence the capability of parasitic elements to float. Therefore, the Mini-FLOTAC, as other flotation-based techniques, is affected by these factors (8, 28–30). For these reasons, Standard Operating Procedures recommend standardized preservation protocols: for livestock, feces freshly collected can be preserved up to 3 days, or as yet mentioned, under vacuum up to 21 days, at +4°C. However, fixatives used for preserving human stool samples usually contain formalin which is toxic, therefore special precautions have to be used for personnel's safety (e.g., use of cabinet or masks) and for discarding the analyzed fecal samples (e.g., containers for special hazardous waste).

The flotation solution to be used can also be standardized for specific target organisms (e.g., saturated sodium chloride, specific gravity 1,200 for FEC and FECRT of GINs in livestock) (27). Instead, the detection of eggs of trematodes requires a flotation solution with high specific gravity (e.g., zinc sulfate 1,350) to improve the efficiency of the Mini-FLOTAC technique, however, when the eggs number is very low, centrifugation or sedimentation based techniques are to be preferred for flukes (8).

To obtain accurate results, as for other diagnostic parasitological techniques, it's important that the technicians have experience to recognize the parasitic elements and to distinguish them from air bubbles, artifacts or pseudo-parasites (31).

Finally, a main weakness is that, at the moment, the distribution of the Mini-FLOTAC and Fill-FLOTAC is managed only by PAR-UNINA and this could limit the commercialization of the devices.

#### Opportunities

The strengths of the Mini-FLOTAC and Fill-FLOTAC offer different opportunities for their commercialization.

The international demand of standardized diagnostic techniques to manage and control parasites in Europe is growing, as well as the requests from research institutes, universities, diagnostic laboratories, veterinary clinics, veterinarians and farmer associations. For these reasons, it could be fundamental to create a network between the above-mentioned stakeholders and the University of Naples to expand the Mini-FLOTAC technique in the international market.

All the information to purchase the Mini-FLOTAC and Fill-FLOTAC devices is available on the website www.parassitologia.unina.it. Through this system, it is very easy for the University to give information before and after the purchase. In addition, detailed tutorials are available on the website (https://www.parassitologia.unina.it/flotac/fill-flotac/video/; https://www.parassitologia.unina.it/flotac/mini-flotac/video/) or alternatively on request, free individual or group residential courses are organized at the PAR-UNINA laboratories to be trained on the standardized use of the techniques. These channels provide opportunities to build a community of trained and informed users, and also a route to add value to the method itself through education and dissemination of related skills and knowledge (e.g., in the application of diagnostics to target anthelmintic use and evaluate efficacy).

The PAR-UNINA gives support to veterinary associations, farmers' associations and veterinary clinics for diagnosis of parasitic diseases, through commercial agreements. The research activities at PAR-UNINA on an automated diagnostic version is ongoing. As an example, prototypes of the Kubic FLOTAC Microscope (KFM), an innovative system to automate the Mini-FLOTAC technique are available for research purposes to reduce time of analysis and human errors (also not expert technicians can use the KFM to identify parasitic elements) (Cringoli et al., accepted).

Using a private agreement with PAR-UNINA, some partners in other continents can become distributors of the Mini-FLOTACs and Fill-FLOTACs, buying large numbers of the devices with a discount and reselling them at the same prices as PAR-UNINA. This arrangement provides opportunities for global reach and impact.

#### Threats

The main threat for the creation of a new European market is the difficulty to convince some veterinarians or lab technicians to adopt innovative techniques and change their practices. This applies both to the principle of using diagnostic information in decision-making (i.e., to treat animals only after a diagnosis, as well as to inertia in the methods currently used for FEC, due to operator familiarity with existing methods). For these reasons, it is very important to plan an information network on best practices with the aim also to counteract the possible pressure to keep a low price for diagnosis from the veterinary sector and the misinformation or influence by external factors of the customers. However, the PAR-UNINA, as described above has a strong expertise and can provide, not only the devices, but also technological and assistance support.

Another main concern is the possibility to be copied, even if the devices have been already patented. About this problem the market will be continuously monitored, and if necessary legal action will be taken.

### The Italian PESTEL Analysis of the Mini-FLOTAC and Fill-FLOTAC

The PESTEL analysis that could influence the Italian commercialization of the Mini-FLOTAC and Fill-FLOTAC is presented in Table 3.

**Table 3:** Factors and sources considered for the Italian PESTEL analysis of the Mini-FLOTAC and Fill-FLOTAC.

**PESTEL factors**	**Sources**
Political	- The Government is a democratic republic. The political situation has not influenced the Mini-FLOTAC and Fill-FLOTAC distribution - Regulation of import and export (EU Directive n. 952/2013) - There is a national plan to contain antimicrobial resistance (www.classyfarm.it; www.vetinfo.it), but not anthelmintic-resistance. A pillar of this antimicrobial resistance plan is the introduction of the law 167/2017 to make the use of an electronic veterinary prescription mandatory - There is no sensibilization or pressure from the government or pharmaceutical industry guidelines (recommendations) to use diagnosis before treatment in livestock farms - Low number of projects on parasitological research funded by the Italian government (e.g., PRIN projects) (www.researchitaly.it)
Economic	- Low economic growth - GDP + 0.2% (2019) (www.istat.it) - 22% VAT (Law 99/2013) - Low development of the livestock sector (−0.2% in 2019; www.istat.it) - Low levels of subsidies for farmers (usually at regional level from EU funds, e.g., Rural Development Plan- PSR 2014-2020) - High presence of intensive farming for large ruminants to reduce costs (www.istat.it) - Often the antiparasitic treatments are made without a diagnosis, using broad spectrum anthelmintic drugs - Low interaction among stakeholders, policy makers, small and medium enterprises, universities, and national public research institutes for the development of new diagnostic tools in ruminant diseases
Social	- Population 60,359,546 - Growth rate −0.4% - Birth rate +7.3% - Death rate +10.7% - Mean age 45.6 - Education level—most of the population have at least a high school degree (www.istat.it; 1st January 2020) - Increasing attention for human and animal health and well-being - Mean age of farmers is decreasing and more often the farmers are graduates. For these reasons the new farmer generation is more inclined to change and use new technologies (especially for large ruminants) - Scarce attention of veterinarians and farmers for parasitological diagnosis in livestock - Parasitological diagnosis for livestock is performed mainly at the Universities and National Public Research Institutes - Farmers are not aware of the importance of parasitological diagnosis and an appropriate treatment to increase livestock production - Most veterinarians don't consider the importance to use FEC and FECRT to evaluate the efficacy or resistance of the treatment
Technological	- Technology incentives—financial support from the National Fund for Innovation and R&D activity - Level of innovation—in veterinary medicine the level is increasing, but not in parasitological diagnosis - There are no direct competitors for the Mini-FLOTAC technique in Italy
Environmental	- Recycling is increasing - Support for renewable energy - Increasing attention for eco-friendly materials used for laboratory equipment and devices - Increasing interest to environmental drug dispersion, mainly for antibiotics
Legal	- Animal health and welfare laws (L. 623/1985; L.146/2001; L. 306/2004; L. 17/2007; EU Regulation 1/2005; EU Regulation 1099/2009; Legislative decree 126/2011) - Environmental Protection (L. 349 /1986; L. 3/2001; Decree of the President of Republic 120/2003; Legislative decree 152/2006; EU Regulation 904/2019) - Consumer protection laws (Legislative decree 206/2005; L. 244/2007) - Copyright and patent laws (L. 633/1941; Legislative decree 30/2005; Legislative decree 18/2019) - Data protection laws (General Data Protection Regulation 679/2016) - Anti-trust law (L. 287/90; Legislative decree 3/ 2017) - Electronic veterinary prescription (L.167/2017) - No specific regulations for anthelmintic drugs administration - Laws on drug residues in milk and meat (EU Regulations: 470/2009, 37/2010, 880/2017)

All the above-mentioned factors may strongly influence the commercialization of the Mini-FLOTAC and Fill-FLOTAC in Italy. The most important aspects of each of the six PESTEL dimensions are reported below.

#### Political Factors

In Italy, there is a limited involvement of the government in parasitological problems in livestock farming. This is reflected by a very low number of projects on this topic funded by the Italian Ministries of Health, Research or of Agricultural, Food and Forestry Policies. Usually, most of the funded projects are on cancer, genetic diseases, or bacterial and viral diseases both in public and veterinary health (www.researchitaly.it). Moreover, even though the problem of AR is increasing, only the issue of antimicrobial resistance has received attention from the Italian Ministry of Health. In 2017 a national plan was developed to counteract antimicrobial resistance, based on the development of an information platform for veterinarians (www.classyfarm.it; www.vetinfo.it) (32) and the introduction of the law 167/2017 to make the use of an electronic veterinary prescription mandatory. Although AR is not mentioned in this law, with the mandatory traceability of veterinary drugs, the use of anthelmintics might, in time, become more closely regulated. At the moment, anthelmintics can be purchased without prescription by e-commerce.

#### Economic Factors

From an economic point of view, one negative factor is that the farmers obtain small profit margin and low levels of subsidies from the government and they often believe that the use of broad spectrum anthelmintic drugs, without a diagnosis, is economically advantageous. In actual fact, the presence of helminths and the incorrect use of anthelmintics influence livestock production, with high economic losses for farmers (1), and diagnosis-targeted treatment has the potential to increase production efficiency, but this is not widely appreciated.

Moreover, in Italy, there is a scarce interaction among stakeholders, policy makers, small and medium enterprises, universities, and national public research institutes to create a network aimed at the commercialization of new diagnostic tools, especially in the field of veterinary parasitology.

#### Social Factors

As demonstrated by Vande Velde et al. (33–35), farmers' intention to adopt innovative systems to control GIN, is not based only on rational and economic, but also on socio-psychological factors. Often, the farmers don't have: (i) the perception of the severity and impact of GIN infections, because the animals are mostly asymptomatic; (ii) the knowledge of AR and its effect on livestock production. In farmers' decision-making, the veterinarian's opinion seems to be an important factor. For these reasons it is important to convince firstly the veterinarians, in order to indirectly influence the farmers (domino effect).

A positive aspect for the Mini-FLOTAC and Fill-FLOTAC distribution in Italy is that the mean age of farmers is decreasing and more often they are graduates. For these reasons, the new farmers' generation is more inclined to change and use new technologies (especially for large ruminants). Unfortunately, as mentioned above, there is a scarce attention of veterinarians and farmers to parasitological diagnosis in grazing livestock and to the use of FEC and FECRT to evaluate the efficacy or resistance of the treatment.

#### Technological Factors

One of the positive aspects for the Mini-FLOTAC and Fill-FLOTAC implementation and development is that in Italy there is financial support from the National Funds for Innovation and R&D activity. Recently the PAR-UNINA obtained a grant from the Ministry of Economic Development to develop a field kit based on the on-farm use of the Mini-FLOTAC technique. In other words, PAR-UNINA promotes the continuous improvement of the Mini-FLOTAC technique, which makes it unique in the market.

Another positive factor for the distribution of the Mini-FLOTAC and Fill-FLOTAC is that the level of innovation in livestock farming is increasing. In fact, Precision Livestock Farming (i.e., the application of process engineering principles and techniques to livestock farming to automatically monitor, model and manage animal production) is now a reality in different settings (36, 37). Based on this strategy, it is fundamental to promote also a “Precision Parasitology,” using best practices of diagnosis and control of livestock helminths also to reduce the environmental impact of livestock farming, which is one of the main goals of the Precision Livestock Farming concept (38, 39).

Another positive aspect is that there are no direct competitors for the Mini-FLOTAC technique in Italy. Usually, easy and cheap diagnostic methods (i.e., fecal smear, simple flotation in tube, flotation in centrifuge) are used by veterinarians, but as described in strengths and opportunities, the Mini-FLOTAC and Fill-FLOTAC can be used either in the laboratory or in the field, obtaining standardized results (8).

#### Environmental Factors

In Italy, as in other parts of Europe, there is increased attention to any product's environmental impact (Law 349/1986; Law 3/2001; Decree of the President of Republic 120/2003; Legislative decree 152/2006; EU Regulation 904/2019). A tax has been introduced this year for plastic single-use items that have the function of containing, protecting or delivering, especially for beverage and food products. For this reason, the development of a biodegradable Fill-FLOTAC is important, but for the moment the Mini-FLOTAC and Fill-FLOTAC, being re-usable, are relatively eco-friendly devices.

#### Legal Factors

In Italy, there is a lot of attention for animal health and welfare (L. 623/1985; L.146/2001; L. 306/2004; L. 17/2007; EU Regulation 1/2005; EU Regulation 1099/2009; Legislative decree 126/2011). Although there are no specific regulations for anthelmintic drug administration, the electronic veterinary prescription that was introduced in 2017 (Law 167/2017) could be useful to reduce the abuse of drugs, including anthelmintics. Moreover, different laws (EU Regulations: 470/2009, 37/2010, 880/2017) regulate the drug residues in milk and meat. For these reasons, best practices of diagnosis and control of livestock infections may be very important and can guarantee also consumer protection.

Regarding new diagnostics, there aren't laws that regulate their use in veterinary medicine. However, the copyright and patent laws (L. 633/1941; Legislative decree 30/2005; Legislative decree 18/2019) guarantee the intellectual property.

Overall, the analysis reported above showed that, in Italy, there are no important barriers for the commercialization of the Mini-FLOTAC and Fill-FLOTAC devices.

### The European PESTEL Analysis on Mini-FLOTAC and Fill-FLOTAC

According to the PESTEL analysis in the 20 European countries involved in the COMBAR WG1, the commercialization of the Mini-FLOTAC and Fill-FLOTAC devices can be evaluated through the 18 variables listed in Table 4.

**Table 4:** Identification of 18 variables from the European PESTEL analysis of the Mini-FLOTAC and Fill-FLOTAC in 20 European countries involved in the COMBAR WG1.

**Dimension**		**Name of variables**	**Concept of variable**	**Keywords**	**Count how many times it appears in the questionnaire**
Political	1	Public policies	Stability of the government	Stable, instable, stability, crisis	10
	2	Awareness policies	If there are awareness policies to use diagnosis before treatment	Regulation, diagnosis, diagnostic methods, anthelmintics, funds, research	13
	3	Anthelmintic strategies	If there is a plan to use anthelmintics	Plan, anthelmintics, parasite control	0
Economic	4	Farming system	Development of farming system	Low, moderate, high	11
	5	Parasitological treatments	Treatments after or without diagnosis	Diagnosis, treatment	7
	6	Network	Interactions among stakeholders, policy makers, small and medium enterprises, Universities and National Public Research Institutes for the development of new diagnostic tools	Stakeholders, policy makers, small and medium enterprises, Universities and National Public Research Institutes	6
	7	Support	If there is support or subsidies to farmers	Farmers, subsidies	5
Social	8	Farmer behavior	If farmers are inclined to change	Farmers, change, innovation, development	7
	9	Diagnosis impact	The importance of diagnosis for farmers	Diagnosis, treatment, animal health	12
	10	Laboratory support	Which laboratories perform parasitological diagnosis?	Laboratories, diagnosis, private, universities	2
Technological	11	Technological Incentives	Are there incentives from national funds?	Incentives, funds, development	7
	12	Innovation	Level of innovation in the farms	Farms, innovation, technologies	13
	13	Competing devices	Are there competitors of the Mini-FLOTAC technique in your country?	Competitors	12
Environmental	14	Eco-friendly device	Attention to use of eco-friendly materials for equipment	Ecology, eco-friendly, environment, green, biodegradable	15
	15	Eco-friendly drugs	Attention to environmental dispersion of drugs	Environment, drugs, dispersion, contamination	2
Legal	16	Veterinary prescription	Regulation of veterinary prescription	Regulation, prescription, treatment	3
	17	Anthelmintic regulation	Regulation for anthelmintics	Treatment, anthelmintics	4
	18	Residues regulation	Laws on residues in milk and meat	Residues, milk, meat	11

The three factors mostly cited from all the interviewed researchers were: (i) the attention to use eco-friendly materials for equipment; (ii) the awareness of and policies to use diagnosis before treatment; and (iii) the level of innovation in the farms. The following sections evaluate the similarities and differences across respondents in each of the factor dimensions.

#### Political Factors

Most of the countries involved in the survey are politically stable. Among all, new regulatory barriers to the importation of diagnostic devices were anticipated as a potential problem only in the post-Brexit UK. Currently, however, there is no registration process to place a veterinary diagnostic device on the UK market, nor any requirement to place a product notification or to follow any specific importation procedures; and so far, there is no intention known to the authors to change this situation.

The lack of awareness, policies and predisposition to use diagnosis before treatment was reported to be common for most of the countries, according to the expert opinions. However, in Europe increased attention is paid on animal health and welfare. For example, Romania has developed policies to protect and support breeders; whilst Poland has taken initiatives for funding of the development of new diagnostic methods. The new political vision of some countries could, therefore, favor the introduction of the Mini-FLOTAC technique.

#### Economic Factors

Regarding the development of the farming system, most of the countries reported a low level, moderate in Germany and high in Poland. The UK highlighted the problem that often drug cost is lower than diagnostic cost, so it is cheaper to treat without using FEC and FECRT. Veterinarians rarely perform FECs themselves, but usually send the samples to veterinary laboratories and the costs are high, as reported also from Ireland and Republic of Serbia. In addition, farmers have few subsidies, usually only from the EU and are not encouraged to invest in diagnosis. Therefore, the introduction of a cheap FEC technique, as the Mini-FLOTAC, and the creation of a network among stakeholders, policy makers, small and medium enterprises, universities and national public research institutes could increase demand. In this way, for the farmers it could be economically advantageous to use a diagnosis strategy before treatment, avoiding the use of ineffective drugs with potentially high production losses, as a consequence.

#### Social Factors

In relation to the importance of diagnosis for farmers, Belgium, Ireland, Italy, Lithuania, North Macedonia, Poland, Portugal, Romania, Republic of Serbia, Slovakia and Spain, considered low levels of consciousness. In contrast, Germany reported a moderate level of consciousness and Austria a high level. In Sweden, the veterinary and farmers' organizations advise farmers to conduct FECs before treatment. The UK reported that often the farmers' behavior is strongly influenced by peers, as much as advisors. The sale of drugs could be correlated also to diagnostic service providers. Moreover, industry-led groups were formed to promote the sustainable control of parasites in sheep (SCOPS; www.scops.org.uk) and in cattle (COWS; www.cattleparasites.org.uk). The core of these groups is composed of different actors: farmers, vets, animal health advisers, parasitologists, researchers, and others. However, the industry bodies in the UK strongly recommend FEC and FECRT for sheep (SCOPS), but not yet for cattle (COWS). Nevertheless, SCOPS is a valid example that a network is efficient to spread the message that diagnosis is very important. Unfortunately, in the UK (as in other countries, e.g., Greece, Italy) accurate FEC and FECRT are not performed in all the laboratories, so it is not easy for a farmer to access this service.

Moreover, a decrease in the age of farmers was registered in Greece as was reported also in Italy (unlike Belgium, Ireland, or Spain). This is a favorable factor, because younger farmers are more inclined to change. However, usually they do not yet have the knowledge to adopt precision strategies on their farms, in relation to helminth control.

For these reasons it would be important to conduct sensibilization campaigns to increase the consciousness and knowledge, not only of farmers, but also of veterinarians regarding the importance of FECs (33–35, 39, 40).

Therefore, as described also above a network between different institutions could be a valid approach to offer a more easily accessible service of diagnosis and to change the current strategy of parasite control in farms.

#### Technological Factors

In general, an inclination to innovation is spreading, also supported by national funds (e.g., in Czechia, Ireland, Italy, Lithuania, North Macedonia, Poland, Spain, and Switzerland). The main limit of the innovations are the costs, because there is the need for low-cost improvements for farmers. The main competitor of the Mini-FLOTAC technique in many countries is the McMaster method. However, different comparison studies performed between these two techniques showed that Mini-FLOTAC method is more sensitive, precise and accurate for the FEC and FECRT in livestock (9, 11–15, 17). In UK there are also other competing methods commercially available, the FECPAK and FECPAK^G2^, providing specific features such as pen-side digital data recording avoiding the requirement of a laboratory or specific technical skills. However, for the moment they have a limited uptake, which may be caused by a relative high cost and low diagnostic sensitivity (10, 41, 42). Nevertheless, any technological contribution leading to increased use of diagnostic tools within the control of helminth infections will help to promote the concept of targeted treatment and thus also the successful market introduction of competing technologies.

#### Environmental Factors

Almost all countries reported that the importance of environmental protection is increasing also in the agriculture field, not only using eco-friendly materials, but also avoiding the dispersion of drugs. In Poland and UK a high interest was developed also for eco-friendly equipment. For these reasons, the use of the Mini-FLOTAC and Fill-FLOTAC could be advantageous, because: (i) they are reusable and so the impact of plastic materials used is low; (ii) a correct and reliable diagnosis could limit the use of treatments, especially if unnecessary, and hence non-target effects in the environment; (iii) there are no greenhouse gas emissions due to sample shipping, when the devices are used on the farm (pen-side use), in contrast to laboratory-based tests.

#### Legal Factors

Almost all the governments of the countries involved in the PESTEL survey have specific laws for environmental protection. Moreover, all the countries have laws for animal health and welfare, to guarantee also the consumer protection; although there are no regulations on the use of anthelmintics, but only on drug residues in milk and meat. A high attention is applied to antibiotics and antimicrobial resistance in different countries. In 2019, an European regulation (EU Regulation 2019/6) on veterinary medicinal products, including antiparasitics, was approved by Parliament in order to limit the risk of development of resistance. However, at the moment, only in some European countries the prescription is compulsory.

#### Other Limitations

No information was reported regarding the implications of the COVID-19 pandemic on Italian and European PESTEL analysis, because the interview was carried out before the European epidemic of SARS-Cov-2. However, the pandemic should be considered for the commercialization of the Mini-FLOTAC and Fill-FLOTAC. The lock-down strategy was the most used approach to control the SARS-Cov-2 transmission worldwide, but as expected from economists there could be a global recession, because most “unnecessary” activities were closed (43). During this period a general decline in the volume of bank transactions and profit, as well as a crisis in financial markets was registered (43). Moreover, it could be considered also the societal costs of COVID-19 measures of restriction that could cause, in the long term, many damages (https://kreavet.com/blog/coronavirus-covid-sars-cov-2-experts-health-policies/). In this chaotic situation, also the research field has completely changed, in fact all now is in the COVID-perspective (https://kreavet.com/blog/animal-health-scientist-point-of-view-on-the-alleged-connection-between-animal-production-and-covid-19/) and many private and public funds may shift priorities to produce diagnostic kits, to find treatments and develop vaccines for COVID-19 (44, 45). In this scenario, universities, public research institutes, but also veterinarians and farmers could have difficulties to obtain funds to buy the Mini-FLOTAC and Fill-FLOTAC. On the other hand, experiences during the COVID-19 pandemic might refocus priorities toward robust food chains, including production; while diagnostic tools that enable accurate estimation of infection with limited or no person-to-person contact (i.e., pen-side tests, are well-placed to support efficient production under distancing criteria), at minimal cost.

## Conclusions

In conclusion, although as reported in the SWOT analysis the Mini-FLOTAC and Fill-FLOTAC show many strengths and opportunities, the main barriers for their commercialization could be related to: (i) political factors, [i.e., in UK, the post-Brexit import regulations (currently unknown)]; (ii) economic, social, and technological factors connected to farmers and veterinarians, because there is a general disinformation or incorrect training, therefore, in many countries there is an inclination to prefer blind treatments vs. diagnosis; (iii) environmental factors are not a limit, because the diagnosis can counteract the indiscriminate use of anthelmintics; (iv) legal factors are not so relevant, because helminth infections do not fall under the regulated diseases. For all these reasons, it is very important to create a network among stakeholders, policy makers, small and medium enterprises, universities and national public research institutes, that acts at the national and European level to get the attention of governments, legislates, veterinarians, farmers, and animal associations on the importance of investing in new efficient and effective diagnostic methods for livestock helminth infections.

Considering the similarities and differences between countries for the six different dimensions as reported in Table 5 and the characteristics of each country, the commercialization of the Mini-FLOTAC and Fill-FLOTAC could be easier in Germany, Poland, Switzerland, and UK (post-Brexit permitting).

**Table 5:** Summary of similar and different countries based on the six market dimensions.

	Exception*	**Similar**	**Different**
Political	UK	All others	Poland, Romania, and Switzerland
Economic		All others	Germany and Poland
Social		Belgium, Ireland, Italy, Lithuania, North Macedonia, Poland, Portugal, Romania, Republic of Serbia, and Slovakia	Austria and Germany
			Greece and Italy
			Sweden and UK
Technological	UK	Czechia, Ireland, Italy, Lithuania, North Macedonia, Poland, Spain, and Switzerland	All others
Environmental		All	
Legal		All	

* *The UK is considered an exception for political factors, caused by the post-Brexit scenario. Regarding technological factors, it is the only country where the FECPAK^G2^ is used at the moment*.

A potential bias of the SWOT and PESTEL analyses performed in this paper was that they included the opinion of researchers that invented the Mini-FLOTAC and Fill-FLOTAC devices. However, since 2013, the Mini-FLOTAC technique has been continuously implemented thanks to swift feedback from researchers from all the world.

The next step aimed at eliminating part of the main barriers for their commercialization and to increase the ability of the Mini-FLOTAC and Fill-FLOTAC to better meet the needs of end-users, could be the development of a Target Product Profile establishing the minimal (the lowest acceptable output for that characteristic) and optimal (the ideal output for that characteristic) characteristics, useful to obtain effective diagnostics (46, 47). The Target Product Profile could involve all the stakeholders, by a survey: in-country and out-country end-users representatives, leading scientists and experts in parasitology, industry scientists, that will contribute to understand if the expected performance, operational characteristics, and costs are able to meet the needs of end-users. Therefore, the Target Product Profile approach will eliminate also the limits of the survey conducted in this paper, that was based only on parasitologists' opinions.

## Data Availability Statement

The raw data supporting the conclusions of this article will be made available by the authors, without undue reservation.

## Author Contributions

MPM, OMDM, and LR conceived, designed the study, and analyzed data collected in the survey. All authors contributed to the survey, preparation of the manuscript, read, and approved the final manuscript.

## Conflict of Interest

The Mini-FLOTAC technique was developed and is patented by GC, but the patent has been handed over to the University of Naples Federico II. The fact that GC is the current patent holder of the Mini-FLOTAC and Fill-FLOTAC had no role in the preparation and submission of the paper. The remaining authors declare that the research was conducted in the absence of any commercial or financial relationships that could be construed as a potential conflict of interest.
